# Predictive Biomarkers of Systemic Therapy Response in Cutaneous Melanoma

**DOI:** 10.3390/cancers18152487

**Published:** 2026-08-03

**Authors:** U Sin Wong, Dajiang Guo

**Affiliations:** 1 Department of Anatomical and Cellular Pathology, State Key Laboratory of Translational Oncology, Prince of Wales Hospital, The Chinese University of Hong Kong, Hong Kong; 2 Li Ka Shing Institute of Health Sciences, Faculty of Medicine, The Chinese University of Hong Kong, Hong Kong

**Keywords:** melanoma, systemic therapy, biomarker, immunotherapy

## Abstract

Late-stage cutaneous melanoma management has already evolved into systemic therapy thanks to the fast development of novel targeted inhibitors and immune checkpoint blockers. Therefore, a reliable prediction of systemic therapy response became crucial for making a personalised management plan for melanoma patients. Although there are multiple biomarkers already available, the overall predictive confidence remains low due to heterogeneous responses among patients. Therefore, novel solid predictive biomarkers are still greatly needed. With the development of new technologies and artificial intelligence, new predictive models are being proposed and generated. In this review, we thoroughly evaluated the established biomarkers that are already in use for clinical practice. In addition, we proposed the emerging new markers with great potential by critically reviewing the literature. Furthermore, we proposed a future framework for making a tailored clinical management plan for melanoma patients.

## Simple Summary

Cutaneous melanoma treatments have been transformed to systemic therapy by the advent of targeted therapies and immune checkpoint inhibitors. However, patient response to melanoma therapies remains highly heterogeneous. To date, many biomarkers have been developed to predict systemic therapy response, but the confidence is low. Novel predictive markers are still greatly needed. In this review, we critically evaluated predictive biomarkers of systemic therapy response in cutaneous melanoma and proposed promising novel markers. Finally, we believe that a practical framework by integrating multi-parametric biomarkers into adaptive treatment algorithms will be the future direction for melanoma management plan making.

## 1. Introduction

The therapeutic strategies for advanced cutaneous melanoma have been transformed by the advent of targeted therapies (e.g., BRAF/MEK inhibitors) and immune checkpoint inhibitors (ICIs). Melanoma management plan-making must take its considerations beyond traditional histology and staging (e.g., AJCC and TNM) and incorporate molecular profiling, most notably BRAF mutation status. According to ASCO guidelines, while surgical excision remains the first-line option for localised invasive melanoma, patients with regionally advanced or metastatic melanoma now benefit from a growing array of systemic therapies that have substantially improved survival [1,2].

Recent clinical trials continue to expand potential systemic therapeutic strategies. Neoadjuvant pembrolizumab has demonstrated high pathological complete response (pCR) rates and favourable safety profiles in resectable advanced melanoma [3,4]. Bispecific antibodies targeting PD-1 and LAG-3 (e.g., tobemstomig) exhibited comparable efficacy to neoadjuvant combination immunotherapy but with reduced toxicity [5]. For *BRAF*-mutant stage III resectable melanoma, neoadjuvant BRAF/MEK inhibition plus PD-L1 blockade resulted in durable recurrence-free survival [6]. In the adjuvant setting, anti-PD-1 immunotherapy (irrespective of *BRAF* status) and BRAF/MEK inhibition (for *BRAF V600E/K* mutations) are now standard of care, with long-term follow-up confirming the sustained benefit of nivolumab over ipilimumab in resected stage IIIB-IV melanoma [7,8,9,10]. For unresectable stage III-IV disease, anti-PD-1 monotherapy remains the preferred first-line approach. However, several novel therapies are under investigation, including different combination regimens, new checkpoint targets (e.g., LAG-3), oncolytic viruses [11,12], and tumour-infiltrating lymphocyte (TIL) therapy (lifileucel) [13]. Emerging strategies such as faecal microbiota transplantation [14,15], aggregable gold nanosystem (Au-TMP) (NCT07562841) and personalised neoantigen vaccines (e.g., BNT221) [16] further illustrate the evolving therapeutic landscape.

Despite these advances, patient responses remain highly heterogeneous. Approximately 35.5% of patients exhibit primary resistance to anti-PD-1 monotherapy, while many initial responders develop acquired resistance, limiting durability [13]. Moreover, immune-related adverse events (irAEs) occur in a significant subset of patients and can necessitate treatment discontinuation [14]. Without robust biomarkers, clinicians risk applying ineffective regimens that may worsen patient condition, delay access to effective alternatives, and expose patients to avoidable toxicity and expenses.

This gap has prompted the development of integrative tools such as the Personalised Immunotherapy Platform (PIP), designed by the Melanoma Institute Australia, which combines multi-dimensional features (i.e., DNA mutations, signalling pathways, immune infiltration, and clinical characteristics) to predict treatment response. Its associated clinical trial, PIP-PREDICT, is currently underway to evaluate predictive accuracy and the potential of biomarker-guided decision-making [17]. An ideal predictive biomarker should be robust, reproducible, cost-effective, and able to precisely distinguish patient subgroups who may benefit or be disadvantaged from specific therapies. Some biomarkers are dynamic, enabling real-time monitoring to guide treatment decisions, adapt therapy before progression or severe toxicity, and potentially assess treatment effectiveness. Established dynamic biomarkers (e.g., circulating tumour DNA, peripheral immune cell ratios, and serial lactate dehydrogenase trends) could partially support adaptive tailoring of treatment plans. In contrast, some biomarkers remain stable throughout the disease course. A classic example is the *BRAF V600* mutation hotspot, which functions as a binary biomarker. Its presence or absence at diagnosis directly informs the effectiveness of BRAFi/MEKi therapy [18]. However, there is still a lack of robust, validated predictive biomarkers, especially for immunotherapies. In addition, the complex aetiology of melanoma and resistance mechanisms, including metabolic rewiring, phenotype switching, and tissue microenvironment (TME) alterations, further highlight the multifaceted nature of the disease and reinforce the need for integrated biomarker strategies to capture its full complexity [19,20].

**Figure 1:**
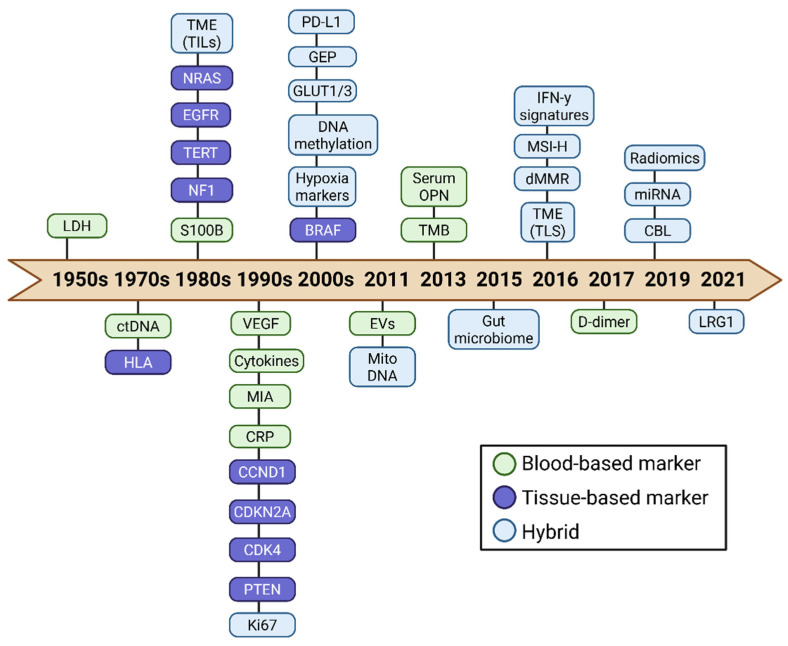
Timeline of biomarker discovery in melanoma, stratified by tissue-based, blood-based, and hybrid categories. The timeline spans from the 1950s through 2021, indicating the year of discovery for each biomarker included in this review. LDH, lactate dehydrogenase; TME, tumour microenvironment; TILs, tumour-infiltrating lymphocytes; MIA, melanoma-inhibiting activity; CRP, C-reactive protein; EVs, extracellular vesicles; Mito DNA, mitochondrial DNA; GEP, gene expression profile; Serum OPN, elevated plasma osteopontin; TMB, tumour mutational burden; MSI-H, microsatellite instability—high; dMMR, DNA mismatch repair deficiency; TLS, tertiary lymphoid structures.

In this review, we critically evaluate biomarkers of systemic therapy response in cutaneous melanoma across three domains: tissue-based markers, blood-based dynamic markers, and emerging candidates (Figure 1). We focus on predictive biomarkers including genetic mutations (e.g., *BRAF*), TME, tumour mutation burden (TMB), gut microbiome, etc. Furthermore, we also discuss prognostic markers which could significantly help predict patient outcome or stratification, such as LDH and s100B. The predictive or prognostic capacities of the markers were summarised in Table 1, application column. We assess their clinical validity, highlight those ready for routine use, and identify gaps requiring prospective validation. Finally, we propose a practical framework for integrating multi-parametric biomarkers into adaptive treatment algorithms, moving from static, one-size-fits-all guidelines toward dynamic, personalised melanoma management that improves overall prognosis.

## 2. Established Biomarkers for Predicting Melanoma Treatments

### 2.1. Tissue-Based Biomarkers

#### 2.1.1. *BRAF* Mutations

BRAF mutations represent the most frequent genetic alterations in sporadic cutaneous melanoma, with V600E being the predominant variant [21]. This substitution constitutively activates the BRAF/MEK/ERK (MAPK) pathway, driving uncontrolled cell proliferation [21]. Other clinically relevant variants include V600K/R/M/D/G, all of which are missense mutations located in the kinase domain [18]. Given its oncogenic nature, *BRAF*-mutated melanoma is generally more aggressive and prone to metastasis compared to *BRAF* wild-type melanoma [21]. From a therapeutic perspective, class I *BRAF* aberrations (V600 mutations) are highly predictive of response to BRAF and MEK inhibitors, whereas class II and III aberrations (non-V600 mutations) confer only modest benefit [22,23]. Christian Menzer and colleagues [24] demonstrated that *V600-nonE/K BRAF* mutations respond better than class II kinase-activating mutations in advanced melanoma. Among both subgroups, the greatest benefit was achieved with BRAFi/MEKi combination therapy. Notably, class II mutations were insensitive to BRAFi monotherapy, while MEKi monotherapy showed comparable responses to the combination regimen [24]. In the CheckMate 067 clinical trial, patients with *BRAF*-mutant melanoma experienced greater benefit from the combination of nivolumab and ipilimumab than from nivolumab monotherapy [25]. Therefore, by stratifying patients according to BRAF mutation status, more informed treatment decisions can be made, potentially reducing exposure to the significant toxicity associated with combined therapies.

#### 2.1.2. *NRAS* Mutations

*NRAS* mutations, most commonly at codon Q61, represent another major driver of melanoma pathogenesis [26]. Notably, they are usually mutually exclusive with *BRAF* mutations, underscoring distinct oncogenic pathways [27]. While *NRAS* mutations lack direct targeted therapies, they are associated with resistance to certain treatments (i.e., MEKi) and thus remain an important biomarker in stratifying patient outcomes [28,29]. Additionally, its mutation revealed clinical benefits in patients receiving BO-112 (particularly without *MYC* amplification) [30].

#### 2.1.3. PD-L1 Expression

Programmed death-ligand (PD-L1) has been extensively studied as a biomarker for immune checkpoint blockade (ICB), particularly anti-PD-1 therapy [31,32]. Functionally, PD-L1 interacts with the PD-1 receptors on T cells and other immune cell populations, causing a reduced anti-tumour immunity [33]. Some studies report that low baseline PD-L1 expression or reduced levels of soluble PD-L1 in serum are associated with improved response and overall survival following ICI therapy [26,30]. Despite biological plausibility, its predictive value in melanoma remains limited. This is partly because PD-L1 expression is highly context-dependent, varying across cellular compartments [34]. Therefore, more promising results have emerged from multiplex immunofluorescence approaches, which integrate PD-L1 with other immune checkpoint markers such as LAG-3, metabolic regulators like iNOS, and melanoma-specific markers (i.e., SOX10) [35]. In the RELATIVITY-047 trial, PD-L1 expression stratified the treatment effect of relatlimab-nivolumab combination versus nivolumab monotherapy in advanced melanoma, regardless of BRAF status [36]. Specifically, patients with PD-L1 ≥ 1% derived no apparent benefit from the combination, whereas those with PD-L1 expression < 1% showed a greater improvement in hazard ratio in favour of the dual regimen, and such benefits were sustained after 4 years [37]. In CheckMate-066 [38] and CheckMate-067 [25] trials, PD-L1 expression was used as a stratification criterion (≥5% vs. <5%). Tumour cell surface PD-L1 positivity correlated with higher response rates to nivolumab in treatment-naïve patients, but PD-L1-negative patients still derived substantial benefit.

Currently, immunohistochemistry (IHC), enzyme-linked immunosorbent assay (ELISA), and immunofluorescence (IF) remain the most widely used techniques for PD-L1 detection. IHC is primarily applied to assess membrane-bound PD-L1, while ELISA enables quantification of soluble PD-L1 [33]. To eliminate inter-laboratory variability, commercial kits such as the FDA-approved PD-L1 IHC 28-8 pharmDx assay (*Agilent*, Santa Clara, CA, USA) have been developed and applied for PD-L1 detection in tumour tissue.

Notably, PD-L1 is not only confined to the tumour cell membrane but also found in the cytoplasm, exosomes, and nucleus [39]. Its expression is dynamic and spatially heterogeneous, which partially explains the unsatisfactory efficacy of current PD-L1 antibodies. Among these compartments, exosomal PD-L1 has emerged as a potentially more reliable biomarker than tumour biopsy PD-L1 expression [33]. Other mutational biomarkers.

Beyond *BRAF* and *NRAS*, several additional mutational events contribute to melanoma heterogeneity and therapeutic resistance. *NF1* loss, frequently associated with high tumour mutational burden, contributes to MAPK pathway dysregulation and confers resistance to targeted therapy [3,40,41]. Similarly, *PTEN* loss impairs PI3K/AKT signalling, correlating with deeper tumour infiltration and poor prognosis [42]. Recently, CBL mutations (deletions at 11q23.1-3) have been identified as new candidate driver events enriched in *NF1* wild-type melanoma [43]. Notably, CBL missense mutations are mutually exclusive with *BRAF* or *RAS* hotspot mutations, while the occurrence of CBL deletion in the *RAS* subtype has been associated with poor prognosis compared with RAS-mutant melanoma lacking *CBL* deletion [43]. Recurrent deletions in *CDKN2A* and *PTEN*, alongside amplifications in *BRAF*, *CCND1*, *TERT*, *MDM2*, and *CDK4*, further underscore the complex genomic landscape of melanoma [43]. Germline HLA status can impact ICI response, with higher evolutionary divergence in HLA alleles associated with better responses [44]. Collectively, these alterations highlight the importance of comprehensive genomic profiling to identify actionable vulnerabilities and stratify patients beyond the canonical BRAF/NRAS framework.

#### 2.1.4. Tumour Microenvironment Profiling

The tumour microenvironment (TME) profoundly shapes immunotherapy outcomes. High pre-treatment densities of tumour-infiltrating lymphocytes (TILs), especially CD8+ cytotoxic T cells, are consistently correlated with improved survival [5,45]. In contrast, immunosuppressive populations such as myeloid-derived suppressor cells (M-MDSCs), M2-type tumour-associated macrophages (TAMs), and regulatory T cells are linked to poor therapeutic response [34]. Interestingly, Foxp3+ regulatory T cells (Tregs) show dual effects. When co-localised with CD8+ T cells, they are linked to resistance to anti-CTLA4 therapy [19]. Meanwhile, some studies demonstrated that the presence of Tregs is correlated with improved overall survival [34].

Metabolic features further modulate immune activity. Tumour glycolysis, mediated by GLUT1/ GLUT3, generally impairs T-cell infiltration. However, T cells located near glycolytic regions often adapt metabolically, acquiring effector phenotypes and secreting IFN-γ [45]. This paradoxical spatial proximity has been linked with improved progression-free survival (PFS), underscoring the importance of host-immune adaptation. Similarly, expression of hypoxia markers (CA9, HIF1α) in CD3+ T cells adjacent to melanoma cells predicts better outcomes [45]. Emerging evidence also highlights metabolic plasticity, including reliance on fatty acid oxidation, as a hallmark of melanoma progression and therapeutic adaptation [43]. Such metabolic traits may themselves serve as biomarkers in the future.

Structural features of the TME add further complexity. The presence of tertiary lymphoid structures (TLSs) has been associated with improved survival after CTLA4 blockade, though variability in TLS number limits clinical applicability [42]. HLA-DR expression has been correlated with differences in response rates to immune checkpoint blockade, while elevated indoleamine 2,3-dioxygenase 1 (IDO1) levels are linked to tumour immune evasion [42]. These conflicting findings highlight the context-dependent nature of melanoma biology. Together, this evidence underscores the importance of integrating spatial, metabolic, cellular, and structural context into composite models for predicting immunotherapy response. Comprehensive TME profiling can be achieved through histopathology, genomics, spatial biology, and radiological imaging.

#### 2.1.5. Tumour Mutation Burden

Tumour mutational burden (TMB) is an established biomarker that reflects the number of somatic mutations per megabase of DNA. High TMB increases neoantigen load, enhances immune recognition, and is associated with improved response rates to ICI therapy [34]. Clinical studies have demonstrated that patients with TMB ≥ 10 mutations/megabase experience significantly longer PFS and OS across treatment groups, including PD-1 monotherapy and combination regimens [4]. Importantly, TMB estimation has proven reproducible across next-generation sequencing (NGS) platforms, supporting its robustness as a biomarker [40]. Dynamic changes in TMB during therapy, such as decreases observed in responders to nivolumab, further highlight its potential as a longitudinal biomarker [46]. In the ongoing BiCaZO trial (NCT05136196), patients are stratified according to TMB and tumour inflammation signatures (TIS) in tumour biopsy (NanoString IO360). These biomarkers are then monitored longitudinally through regular blood tests to evaluate the treatment’s effectiveness between these biomarker-defined subgroups in cabozantinib and nivolumab combination therapy.

DNA mismatch repair deficiency (dMMR) represents a mechanistic driver of elevated mutational burden, giving rise to microsatellite instability—high (MSI-H). MSI-H tumours accumulate replication errors in repetitive DNA sequences, resulting in expanded neoantigen landscapes that enhance tumour immunogenicity. This continuum underpins improved responsiveness to ICI therapy and reinforces their value as predictive biomarkers in melanoma, with TMB serving as a summary measure of mutational load and dMMR/MSI-H representing a mechanistic driver of elevated immunogenicity [47,48]. However, it is worth noting that the prevalence of MSI in primary cutaneous melanoma is not universal, with reported rates ranging from 16.0% to 31.8% and improved rates in metastatic cases [49]. Currently, the DETERMINE project (NCT05770102) in the UK is designed to explore cancer drug repurposing; one of its treatment arms focuses on tumours with high TMB, MSI-H and Constitutional Mismatch Repair Deficiency (CMMRD). The aim is to determine whether these features can be applied to stratify melanoma patients and identify those who may benefit from atezolizumab or even other established cancer drugs in the future.

### 2.2. Blood-Based Biomarkers

Blood-derived biomarkers offer a convenient and minimally invasive means of monitoring disease progression and therapeutic response in melanoma. They are particularly valuable for capturing intra-tumoural heterogeneity that may not be evident in single-site tissue biopsies. They are superior to tissue-based analytes because tissue-based evaluations are usually limited by representing a single time point and spatial assessment, failing to capture patient heterogeneity and inter-/intra-tumoural differences [50].

#### 2.2.1. Lactate Dehydrogenase (LDH)

LDH is a longstanding biomarker in melanoma and can be subdivided into five isoenzymes, namely LDH-1 to LDH-5 [51]. Since melanoma cells rely heavily on glycolysis, LDH plays a crucial role in their energy metabolism [52]. In the human body, elevated LDH levels generally reflect higher tumour burden and rapid cell turnover [53]. High serum LDH indicates widespread cell death or necrosis involving tumour, immune, and normal cells, which often occurs when tumours outgrow their blood supply. This process contributes to an inflammatory and immunosuppressive environment that diminishes immunotherapy efficacy [54].

Clinically, high LDH is consistently associated with shorter PFS and poorer overall survival (OS) in patients receiving systemic therapy [55,56]. LDH is incorporated into the AJCC staging system as an independent adverse predictor of survival in stage IV melanoma [57]. Elevated serum LDH at baseline followed by rising LDH has been observed in progressive disease during treatment with nivolumab or pembrolizumab [58]. In contrast, normal baseline LDH and low TMB predict longer PFS and OS in patients treated with vemurafenib plus cobimetinib [59].

However, LDH suffers from poor specificity, as elevations can occur in diverse non-malignant conditions, limiting its utility as a standalone marker [53]. Also, LDH is not sensitive enough to detect in-transit metastases [60].

Detection relies on indirect measurement of substances involved in LDH redox reactions. Techniques, including colorimetry, UV-spectrophotometry, electrochemical measurement, and fluorometric measurement, can be applied in LDH detection [52]. In summary, LDH reflects tumour aggressiveness and prognosis, and its role is best defined as a dynamic monitoring and prognostic tool.

#### 2.2.2. S100B

S100B is a protein specifically released by melanoma cells and demonstrates greater sensitivity than LDH in tracking disease progression [61]. Baseline elevations of S100B reflect aggressive melanoma biology and predict impaired outcomes in metastatic patients treated with BRAF inhibitors [56]. Importantly, dynamic changes in S100B levels correlate with treatment response: declines after therapy reflect tumour shrinkage, while subsequent rises often precede radiological evidence of progression [62]. This makes S100B particularly valuable for early detection of progression and for close monitoring during targeted therapy.

Despite its promise, several factors prohibit the clinical implementation of S100B [63]. First, it has a short half-life in serum. Second, although its tissue specificity is better than LDH, its elevations are still not restricted to melanoma and can result in false positives. Third, there is no standardised or validated cut-off value; together with the absence of assay standardisation further reduces the reproducibility across studies. Without sufficient validation and proven benefit, its clinical application is not adopted.

#### 2.2.3. Circulating Tumour DNA

Circulating tumour DNA (ctDNA) has emerged as a powerful, non-invasive biomarker in melanoma, offering distinct advantages over conventional protein biomarkers like LDH and S100B. Its major advantage lies in providing a real-time snapshot of tumour burden and heterogeneity, complementing tissue-based assays. This capability is particularly valuable for monitoring tumour dynamics, minimal residual disease (MRD), treatment response, and identifying emerging resistance mechanisms [64].

Baseline ctDNA levels and dynamic changes carry significant predictive weight. For instance, high pre-treatment ctDNA levels have been correlated with lower overall response rates to BRAFi/MEKi therapy [65]. Conversely, BRAF-mutated patients with undetectable BRAF-mutation-positive plasma ctDNA prior to treatment showed higher response rates to dabrafenib and trametinib [66]. CtDNA also helps distinguish true progression from pseudoprogression in immunotherapy-treated patients, as favourable ctDNA profiles are often seen in pseudoprogression, whereas rising levels indicate genuine progression [67]. Meanwhile, mixed responses (some lesions shrink while others grow) can also be captured [68]. Mutation-specific ctDNA assays further enable early detection of acquired resistance mechanisms, such as NRAS mutations in patients treated with BRAFi or activating mutations in bypass pathways (e.g., MAP2K1, AKT1, and PIK3CA), allowing timely adaptation of treatment plans [21].

Additionally, ctDNA has shown promise in detecting MRD and relapse, often outperforming imaging in early detection [5,69]. In advanced melanoma, sensitivity for detecting BRAF-mutation positivity can exceed 75% [66]. Meanwhile, the sensitivity for a 12-month relapse prediction is 55% [70]. A meta-analysis also reported a pooled sensitivity of 73% [71]. Therefore, regular ctDNA monitoring has been applied in adaptive therapy strategies. For instance, a clinical trial is evaluating the impact of continuous versus intermittent combination treatment with encorafenib and binimetinib on resistance development in late-stage cutaneous melanoma [72]. Another ongoing clinical trial (UK DETECTION) is evaluating the impact on OS in standard adjuvant therapy compared with a react-only-after-clinical-relapse strategy (i.e., ctDNA detection or local recurrence) [73]. Ultimately, the community aims to prevent overtreating patients who are unlikely to derive benefit from standard (neo)adjuvant therapy.

Despite its promise, ctDNA detection sensitivity remains a major challenge, particularly in early-stage disease or after curative treatment, where mutant allele fraction may fall below 0.1 [74,75]. Additional barriers include assay variability, lack of standardised interpretation of longitudinal dynamics, lack of standardised validation pipeline and benefit assessment, patient-related factors (i.e., diet), and tumour heterogeneity. In addition, its sensitivity can be influenced by anatomical location of relapse, total number of metastatic sites, and sampling timing (based on treatment applied) [70].

To overcome the sensitivity limitations, several methodological advances have been developed. CtDNA fragments are typically shorter than cfDNA from normal cells, facilitating discrimination. Common detection platforms include Digital Droplet PCR (ddPCR) and NGS. Recent advances in amplification strategies, such as TOP-PCR, have improved sensitivity [74]. This half-adapter ligation design and single-primer PCR enable cost-effective pre-amplification compared to NGS. Longitudinal sampling enhances detection rates and reduces false positives, especially in the post-surgical setting where the sensitivity is poor [76].

TMB derived from ctDNA serves as an alternative to tissue-based TMB, while MSI detected in ctDNA has been linked to a vigorous immune microenvironment and enhanced response to ICI therapy [77]. NGS-based ctDNA-MSI approaches are becoming more sensitive and concordant with tissue MSI status. MRD assays can be tumour-agnostic and tumour-informed, the latter requiring prior knowledge of tumour genomic alterations. Best practices in sample collection and analysis are gradually being discussed and standardised, with Streck tubes and plasma isolation increasingly adopted to reduce contamination [78,79,80]. FDA-approved commercial kits such as FoundationOne Liquid CDx (*Foundation Medicine*, Boston, MA, USA) and Guardant360 CDx (*Guardant*, Palo Alto, CA, USA) are also emerging [80]. Ultimately, ctDNA testing in conjunction with imaging and clinical examinations may overcome limitations where metastatic sites are inaccessible.

#### 2.2.4. Peripheral Blood Biomarkers

Beyond ctDNA, peripheral blood immune ratios provide accessible predictive insights. Elevated neutrophil-to-lymphocyte ratio (NRL) is independently linked to non-response to immunotherapy, while low lymphocyte-to-monocyte ratio predicts poor outcomes [81]. Higher absolute counts have correlated with favourable immunotherapy response in some cohorts, whereas platelet-to-lymphocyte ratio (PLR) and systemic immune-inflammation index show modest utility [82]. Baseline serum levels of eosinophil activity marker (EPX), eosinophil cationic protein (ECP) and hypercholesterolemia have been associated with better response to ICIs. White cell blood count and haemoglobin levels are also useful indicators for ICI therapy response [83]. These ratios are attractive due to their accessibility and low cost, though their role remains in monitoring response and prognosis rather than informing initial therapy choice. Apart from efficacy prediction, accurate risk assessment for potentially life-threatening toxicities is critical. For instance, a low baseline proportion of peripheral blood CD4+ Tregs has been linked to subsequent colitis caused by ipilimumab [84]. A baseline absolute eosinophil count > 240/μL or a relative eosinophil count can predict PD-1-related endocrine toxicities [85]. Low baseline NRL and PLR are independent predictive markers for ICI-related toxicities [86]. Early changes in B cells after combined treatment predict higher rates of high-grade toxicities [87]. High baseline and early increases in pro-inflammatory cytokines, including IL-1a, IL-2, and IFN-a2, are observed in patients with severe toxicities [88].

Inflammatory and coagulation markers add complementary information. Elevated C-reactive protein (CRP) and D-dimer reflect systemic inflammation and coagulation activity, respectively [89,90]. Peripheral immune cell profiles, such as absolute neutrophil counts and circulating CD3+ T cells, have also been explored. For example, CD8+CD45RO+ effector memory T cells correlate with longer survival under CTLA-4 blockade, while elevated neutrophil counts may reflect adverse treatment effects [34]. Elevated baseline levels of tumour-derived extracellular vesicles (EVs) have been linked to poor prognosis and immunotherapy resistance [42].

Disruption of cell-to-cell interactions, quantified as melanoma-inhibiting activity (MIA), facilitates migration and metastasis [91]. Elevated serum MIA levels are associated with a higher risk of recurrence and disease progression, whereas they decline significantly after BRAF inhibitor treatment. High serum Vascular Endothelial Growth Factor (VEGF) levels also predict lower response rates to ipilimumab and overall reduced survival [91].

#### 2.2.5. Extracellular Vesicle-Based Biomarkers

EVs carry DNA, RNA, proteins, metabolites, and lipids, mediating intercellular communication within the TME. This information can be profiled using qPCR, NGS, or microarray techniques [92]. Their abundance in biofluids, comparative stability conferred by lipid bilayers, and long-term storage potential at −80 °C make them attractive for minimally invasive biomarker discovery [93]. EVs are heterogeneous, encompassing exosomes, microvesicles (ectosomes), large oncosomes, and apoptotic bodies [93]. In melanoma, studies have shown that EVs like exosomes can promote cancer progression and become potential biomarkers or therapeutic targets [94,95,96,97]. In addition, scientists have shown that EV-associated PD-L1 induces immunosuppression and promotes tumour growth, while specific EV miRNAs affect treatment outcomes and resistance [93,98].

Exosomal circRNAs, highly stable and abundant in body fluids, represent novel diagnostic and prognostic biomarkers. They facilitate tumour–microenvironment communication and influence cancer progression [99].

#### 2.2.6. Plasma Proteomic Biomarkers

Elevated plasma osteopontin (OPN) levels are significantly associated with metastatic disease, advanced AJCC stage, and increased probability of progression [91,100].

## 3. Limitations of Current Biomarkers

The clinical utility of predictive biomarkers in melanoma is constrained by multiple technical, biological, and translational limitations. Tissue-based biomarkers such as TMB suffer from sampling error, reliance on small biopsies, hypermutated tumours, and variability across sequencing panels, all of which undermine reproducibility and predictive accuracy [50]. Similarly, PD-L1 assays face high inter-assay variability due to differences in antibody clones, staining thresholds, and scoring systems, while expression varies between primary and metastatic sites, complicating interpretation [47].

Blood-based biomarkers also present challenges. ctDNA is limited by low levels in early-stage or non-shedding tumours, short half-life, contamination from clonal haematopoiesis, and lack of standardised cut-offs [67]. Methodologically, ddPCR offers precision but is restricted in multiplexing (pre-designed primers), whereas NGS expands scope but increases cost (cost will decrease in the future) and may reduce accuracy [101]. EVs face hurdles in purity, quantification, and methodological standardisation, particularly in extraction protocols [59]. Protein biomarkers such as LDH and S100B lack specificity, as elevations can occur in non-malignant conditions, while detection methods such as colorimetry or UV-spectrophotometry are prone to interference [52,92].

Similarly to many other cancer types, melanoma suffers from spatial and temporal heterogeneity. Spatial heterogeneity arises from intra-tumour variability, driven by subclone formation and diversity within the TME, which can amplify the consequences of sampling bias. Meanwhile, temporal heterogeneity reflects fluctuations in biomarker levels over time, influenced by treatment exposure, changes in disease stage or progression, physiological status, and even sudden lifestyle modifications. Collectively, these factors challenge biomarker reproducibility and clinical implementation. Repeated sampling may help compensate for such variability, although financial constraints remain a key limiting factor [102].

When comparing blood- and tissue-based biomarkers, blood-based markers are less sensitive and may capture more background noise, but they can capture the systemic tumour heterogeneity by integrating signals from different lesions. They are also more accessible and feasible for regular sampling, making them practical for longitudinal monitoring. However, blood-based biomarkers only become detectable once tumour-derived molecules are released into the circulation, which may delay their reflection of tumour biology. Tissue-based biomarkers provide more tumour-specific signals, even though these can be diluted by normal cells or confounded by clonal heterogeneity. Importantly, tissue assays are limited to represent only the sampled region, and repeated sampling is less feasible in clinical practice, especially in metastatic cases where biopsies are inaccessible.

Imaging-based biomarkers through radiomics and spatial profiling encounter barriers, including heterogeneous image acquisition, segmentation errors, and inter-reader variability, which limit reproducibility and clinical acceptance [74]. Additional challenges include variability in lesion selection (single vs. multiple), region of interest (intra-tumoral, peri-tumoral, or whole image), and imaging time-points (baseline vs. follow-up, or delta radiomics) [73]. Analysing only one lesion may fail to capture the full spectrum of tumour heterogeneity, particularly in metastatic patients.

More broadly, translational barriers hinder biomarker adoption. These include high cost, labour-intensive interpretation, lack of independent validation datasets, and inconsistent clinical cut-off values. Many gene expression profiling assays have been criticised for selection bias and overlapping patient populations, further limiting their clinical reliability [9,75].

## 4. Emerging Biomarkers

Despite advances in biomarker development, currently available predictive biomarkers remain of limited clinical utility. In this section, we highlight emerging biomarkers that may help explain the heterogeneity of treatment response and resistance in melanoma.

The aetiology of cutaneous melanoma reflects a complex interplay of genetic, epigenetic, and environmental factors. Melanoma harbours the highest mutation frequency among human cancers, driving significant intra- and inter-tumoural heterogeneity [103]. Another defining feature of melanoma is its pronounced cellular plasticity, wherein tumour cells dynamically adapt to microenvironmental cues and transition between various transcriptional states. This plasticity underlies treatment outcomes, as melanoma cells can shift between differentiated and dedifferentiated phenotypes, influencing both sensitivity and resistance to therapy.

In general, established resistance mechanisms can be broadly categorised into genomic, epigenetic, metabolic, and immune alterations. These mechanisms are deeply interconnected and often co-exist within the same tumour. Genomic alterations frequently provide the primary drivers of resistance, with commonly mutated genes including *CDKN2A*, *RB1*, *PIK3CA*, *AKT3*, *MAP3K8*, and *MITF* [103]. Such mutations sustain continuous activation of the RAS/RAF/MAPK, PI3K/AKT, or JAK/STAT pathways. In the context of BRAFi/MEKi therapy, resistance can also arise from *BRAF* amplification, alternative splicing, and *BRAF-CRAF* heterodimerisation, all of which compensate for the blocked MAPK pathway [104]. For immunotherapy, loss of MHC expression caused by B2M mutations, along with reduced expression of lineage antigens (i.e., *MITF*), promotes immune evasion [20]. Epigenetic modification further enables phenotypic plasticity, often driven by microenvironmental signals. Disruption of *PTEN*, *MGMT*, *EZH2*, and *RAR-β2*, along with silencing of interferon transcription, are frequently observed [104]. MicroRNAs such as miR-211, miR-410-3p, and miR-222 also play key roles by activating MAPK/PI3K pathways while simultaneously suppressing dendritic and NK cell function. On the other hand, metabolic reprogramming supports tumour survival under therapeutic pressure by increasing glycolysis, glutamine metabolism and mitochondrial activity. These metabolic shifts lead to microenvironmental acidification, which in turn accelerates resistance development [104].

Accumulating evidence emphasises the contribution of cell state heterogeneity and plasticity in therapeutic resistance development [105,106]. Therefore, reliance on a single marker is insufficient to capture the full spectrum of disease behaviour. This has led to increasing efforts to integrate multiple biological layers into machine learning (ML) models, which can aggregate diverse features to improve the prediction of treatment response. Given the inherent complexity of melanoma biology, ML-based approaches represent a promising avenue for developing a more robust and clinically relevant predictive framework.

### 4.1. Immune-Related Transcriptomic Biomarkers

Gene expression profiles (GEPs) capture immune response phenotypes beyond mutational drivers. Tools such as IMPRES, IFN-γ signatures, and TIDE have been applied to predict ICI response, while commercial assays like Merlin, DecisionDx-Melanoma, and Melagenix estimate recurrence and survival in early-stage disease [22]. Importantly, melanoma subtype variability is notable. For instance, desmoplastic melanoma demonstrates particularly favourable outcomes in anti-PD-1 immunotherapy compared to other forms [26].

Loss of MHC-I expression correlates with ICI resistance, whereas MHC-II expression predicts anti-PD-1 benefit [20]. A combined HLA-I/II expression in tumour has also shown predictive value on anti-PD-1 response [107]. Resistance programmes often involve low CIITA transcripts, loss of heterozygosity across MHC loci, or de-differentiated phenotypes lacking B2M, raising the potential of incorporating these features as specific resistance indicators [20].

Cell state heterogeneity adds another layer of complexity. Transcriptomic profiling has identified melanocytic, transitory, neural crest-like, and undifferentiated states, with the latter two enriched in immunotherapy-resistant tumours [105]. These resistant phenotypes often display pro-inflammatory signalling (IL6, TNFα) and reduced PD-1+ CD8+ T cells, underscoring the interplay between cell differentiation, immune evasion, and therapeutic resistance [106].

Additional transcriptomic markers have been linked to clinical outcomes. Elevated IDO and FoxP3 expression correlate with improved response to ipilimumab, while high Ki67 expression predicts shorter median OS in patients treated with vemurafenib monotherapy but not in combination regimens [59]. Non-responders to neoadjuvant ICI therapy exhibit higher levels of LRG1, indicating poor prognosis [59]. Moreover, EGFR expression has been implicated in mediating resistance to BRAF inhibitors [44].

Epigenetic regulation, particularly DNA methylation, is closely linked to oxidative stress and immune modulation in cancers [108]. Abnormal DNA methylation is a nearly universal feature of melanoma, with progressive hypermethylation linked to tumour aggressiveness [92]. FDA-approved methylation tests (i.e., Epi proColon for colorectal cancer) are already used in other cancers, and emerging evidence shows predictive value in melanoma [109].

The EpiMelanoma test, which analyses cfDNA from plasma, has demonstrated high sensitivity and specificity, with the melanoma-specific region *SPTBN1* (cg04652957) achieving 100% sensitivity and 90.91% specificity [110]. Beyond commonly methylated genes (ABCB1, CHFR, HIST1H4F, ITGA4), recent studies identified 35 differentially methylated positions (DMPs) (responders vs. non-responders) significantly correlated with gene expression, 13 of which were validated across independent datasets, reinforcing reproducibility [111].

DNA methylation regulates melanoma cell immunogenicity, modulates MHC expression and immune checkpoint molecules, and shapes T-cell phenotypes within the tumour microenvironment. For example, methylation of NLRC5 is linked to loss of MHC Class I gene expression, contributing to immunotherapy resistance [112].

### 4.2. miRNA and Non-Coding RNA Biomarkers

Alterations in non-coding RNAs, particularly microRNAs (miRNAs), contribute to melanoma initiation, drug resistance, and immune modulation, making them accessible biomarkers with emerging predictive value [113]. miRNAs regulate gene expression by binding to mRNA, and their stability in plasma, serum, cerebrospinal fluid, and urine makes them readily accessible [114]. Low expression of miR-150, miR-15b, and miR-23a is linked to worse prognosis, while specific miRNAs (miR-16-5p, miR-17-5p, miR-20a-5p) independently predict PD-1 inhibitor response [114,115].

Dynamic changes in the miRNAome (e.g., miR-4443, miR-199b-5p, and miR-4488) discriminate BRAF/MEK inhibitor responders from non-responders, while miR-146a, miR-155, and miR-125b in extracellular vesicles correlate with myeloid-derived suppressor cell expansion and resistance to ipilimumab and nivolumab [114]. Despite these insights, currently, very few miRNA-based diagnostic tools are clinically approved. A few applications are implemented in clinical laboratory settings, including ThyraMIR v2 for thyroid nodule diagnosis [116].

### 4.3. Mitochondrial Genetics

Inherited mitochondrial genetics have revealed predictive power for ICI efficacy in metastatic melanoma [117]. For example, haplogroup T is associated with no clinical benefit from nivolumab as a single agent or combined with anti-CTLA-4, serving as an independent predictor. In addition, mitochondrial pathways (e.g., oxidative phosphorylation (OXPHOS) and mitochondrial translation) are critical drivers of melanoma progression and therapy resistance [118]. For instance, OXPHOS upregulation was found in BRAF-mutant and metastatic tumours, while treated metastases exhibited consistent upregulation of mitochondrial translation machinery and OXPHOS due to shared metabolic rewiring under therapeutic pressure.

### 4.4. IFN-γ Signatures

Interferon-gamma (IFN-γ) is a central cytokine in melanoma, acting as both a predictor of immunotherapy response and a mediator of resistance. It plays a complex, dual role. Elevated baseline serum levels of IFN-γ, alongside IL-6 and IL-10, correlate with favourable outcomes in ICI, while soluble CD73 and persistent IL-8 signal are associated with poor response [34]. Notably, non-responders show 3–8x higher IL-6 levels compared with responders, both pre-treatment and during therapy with ICB alone or in combination with targeted agents in stage IV melanoma [119]. IL-8 levels correlate with tumour size, stage, and survival rates, and may predict response to BRAF inhibitors and immune-targeting monoclonal antibodies [91]. For example, increasing IL-8 serum levels during anti-PD-1 treatment have been linked with positive response, whereas elevated serum baseline IL-8 levels predict poor outcome in ICI-treated patients [120,121]. High IL-17A levels in blood indicated a stronger global TH17 cytokine profile preceding clinical response to dual ICI [122]. IL18 expression is strongly correlated with CD8+ T and NK cell infiltration [123]. Circulating B cells that produce TNF-alpha and IL-6 are associated with unresponsiveness to the anti-CTLA4 antibody [124]. Gene expression profiles enriched for IFN-γ-regulated transcripts consistently predict clinical benefit from PD-1 blockade, underscoring its role as a functional biomarker of immune activation [109].

Neoadjuvant studies using anti-PD-1 and anti-CTLA-4 therapy further highlight its relevance: patients achieving major pathological response (≤10% viable tumour cells) demonstrated upregulation of pro-inflammatory cytokines and immune modulatory markers (HAVCR1, MLN, PRSS8, MME), whereas non-responders upregulated immune-suppressive molecules (ICA1, SCG3) [125]. These findings link IFN-γ-associated immune activation to a durable pathological response.

At the same time, IFN-γ signalling contributes to resistance. Tumour-intrinsic changes such as antigen loss, dedifferentiation, and reduced MHC-I expression enable immune escape despite pathway activation [20]. Resistant lesions often lack PD-1+ CD8+ T cells, tertiary lymphoid structures, and B2M, reflecting the complex interplay between IFN-γ signalling, immune suppression, and tumour adaptation [19].

Mechanistically, high nuclear IRF1 induced by IFN-γ correlates with improved PFS under anti-PD-1 therapy, while a higher IFN-γ/IL-10 ratio in peripheral blood predicts better response [105]. Conversely, prolonged IFN-γ signalling can drive epigenetic and transcriptomic changes that reduce immune infiltration and promote resistance, particularly to CTLA-4 blockade combined with radiation. Mutations in *IFNGR1*, *JAK1*, and *JAK2* have also been associated with primary or acquired resistance to checkpoint inhibitors, rendering melanoma cells insensitive to IFN-γ’s effects.

Clinically, a strong IFN-γ signature combined with high PD-L1 expression and CD8+ infiltration correlates with longer progression-free and overall survival in metastatic melanoma, including patients treated with atezolizumab [59]. More broadly, cytokine dynamics within the TME, spanning pro-inflammatory mediators (IL-6, IL-8, TNF-α, IFN-γ) and immunosuppressive factors (IL 10, TGF-β), capture evolving tumour immune interactions and may better predict treatment outcomes than static baseline measurements [126]. Notably, low intratumoral IFN-γ signatures are associated with poor benefit from neoadjuvant ipilimumab plus nivolumab in stage III melanoma [127].

In the DONIMI trial (NCT04133948), patients were stratified based on baseline IFN- γ signature expression algorithm [127]. Patients with low intra-tumoural IFN-γ signatures were less likely to benefit from standard neoadjuvant ICB. In contrast, patients with high IFN-γ signatures demonstrated markedly higher pathologic response rates to nivolumab monotherapy, allowing differentiation from individuals who require more intensive combination approaches. Emerging clinical trials (e.g., NCT07562841) begin to assess the treatment-related dynamic changes in peripheral blood immune microenvironment, including IFN-γ, TNF-α, and IL-6.

### 4.5. Gut Microbiome

The gut microbiome has emerged as a critical determinant of immunotherapy response in melanoma, shaping both systemic immunity and intrinsic tumour biology. Beneficial taxa such as Bacteroides fragilis (Bfr), Bacteroides vulgatus (Bvu), and Clostridium perfringens (Cpe) produce short-chain fatty acids (SCFAs) that enhance antigen-presenting cell function, activate inflammasome pathways (e.g., NLRC4/IL-18), and suppress Treg differentiation, thereby promoting effector T-cell infiltration [128]. In contrast, Akkermansia muciniphila, Bifidobacterium bifidum, Clostridial cluster IV, and Ruminococcus spp. would drive Treg induction, mucin degradation, and chronic low-grade inflammation, leading to immune exhaustion and PD-L1 upregulation.

Clinical studies reinforce these associations. Patients with Ruminococcaceae-dominated microbiomes respond more favourably to ICIs compared to those with Bacteroidaceae-dominated microbiomes, who also exhibit elevated baseline CRP and poorer outcomes. Diet plays a modulatory role, with fibre and omega-3 intake enriching beneficial taxa [90]. Functional analyses show that butyrate production, B-vitamin synthesis, and amino acid metabolism are enriched in responders, whereas non-responders display reductions in these pathways alongside increased extracellular matrix remodelling and stromal changes. Importantly, microbiome composition also correlates with TMB, IFN-γ signalling, and stromal architecture, underscoring host–tumour–microbe interactions that extend beyond systemic immunity [90].

Microbiota bridge innate and adaptive immunity by releasing metabolites that directly activate T lymphocytes, modulate intestinal barrier integrity, or act on tumour cells. For example, high circulating L-arginine levels are associated with improved PFS in ICI-treated patients [129]. Specific taxa have been linked to reduced irAEs and enhanced ICI response. Conversely, Bacteroides species may increase irAEs, particularly colitis. This phenomenon of efficacy–toxicity coupling highlights that increased risk of irAEs can coincide with heightened ICI sensitivity. Protective metabolites such as indole-3-carboxaldehyde (3-IAld) may mitigate ICI-induced colitis by preserving intestinal barrier integrity [130]. Notably, depletion of Akkermansia muciniphila, Faecalibacterium prausnitzii, and members of the Ruminococcaceae family has been observed in patients with severe irAEs [131].

### 4.6. Radiomics

Radiomics applies AI algorithms to imaging modalities such as CT, MRI, PET, and ultrasound, offering non-invasive biomarkers that capture organ-level heterogeneity and predict treatment response. Pre-treatment CT scans can quantify 3D body composition, with features such as L5 bone volume, L5 subcutaneous adipose tissue volume, pelvis visceral adipose density, neutrophil-to-lymphocyte ratio, and age ranking among the most important predictors in ML models for advanced melanoma [132]. This approach may overcome limitations of blood-based biomarkers, particularly where the blood–brain barrier restricts systemic readouts. CT can also measure low muscle attenuation, which acts as an independent factor associated with high-grade ipilimumab-related toxicities in metastatic melanoma [84]. Radiomics also highlights organ-specific resistance to treatment. MRI and CT features reveal substantial intraorgan heterogeneity in ICI response, especially in the brain, underscoring the dependence of ICI efficacy on local immune microenvironments [133]. While absent in BRAF-treated patients, radiomics may help patients distinguish between ICI and BRAF/MEKi therapy, identify resistant sites for localised therapy, and predict organ-specific outcomes. 18F-FDG PET-CT features such as metabolic tumour volume (MTV) and long zone emphasis (LZE) stratify risk before anti-PD1 therapies, potentially guiding more aggressive treatment strategies [134]. Advanced MRI techniques further expand radiomics applications. Multiparametric MRI (mpMRI) enables early assessment of intratumoural and intertumoural heterogeneity. DCE-MRI evaluates tumour vascularisation, while CEST-MRI detects metabolites at low concentrations with high resolution. Diffusion-weighted imaging (DWI) assesses tumour cellularity, and SWI detects small haemorrhagic areas. Novel probes such as SPIO nanoparticles and 19F perfluorocarbons (19F PFCs) allow MRI tracking of immune cells, while time-dependent diffusion (TDD) distinguishes immune cell populations by estimating average cell size [135].

Radiomics also enables assessment of intra-patient, inter-lesion heterogeneity, identifying non-responding lesions that may require localised treatment [136]. PET/CT scans track metabolic activity and can detect acquired resistance early, guiding therapy adjustments [137]. The established and emerging biomarkers were summarised in Table 1.

## 5. Future Directions

The next phase of biomarker research in melanoma is shifting towards integrated, multi-modal frameworks that are both scientifically robust and clinically achievable. Individual biomarkers rarely provide sufficient predictive power when used alone, especially given the expanding range of therapeutic options. A practical strategy is to combine complementary domains into composite models that balance accuracy, reproducibility, cost, and turnaround time, with the ultimate goal of guiding treatment decisions and improving patient outcomes, while potentially revealing novel therapeutic targets.

Conventional statistical approaches such as hazard ratios and odds ratios are widely used for biomarker validation because they are interpretable and familiar to the clinical community [138]. However, they are limited to handling high-dimensional data. In contrast, machine learning and deep learning approaches can integrate multi-omics datasets and capture complex or potentially hidden relationships (i.e., knowledge graphs) [139]. But they are less interpretable, computationally expensive, and require large, consistent datasets for model construction and later training.

Tissue-based biomarkers provide mechanistic insight but suffer from variability in sampling, assay protocols, end-point assessment method (i.e., PFS, ORR, OS or tumour shrinkage), and thresholds. Standardisation of pre-analytical handling, harmonisation of IHC/assessing platforms, and adoption of digital pathology with AI-assisted scoring are essential to reduce inter-observer variability and enable reproducible interpretation across centres. AI-based anomaly detection for histological images has been emerging across different diseases [140].

Blood-based biomarkers offer minimally invasive monitoring, longitudinal monitoring, and capture of systemic heterogeneity. Cost-effective detection platforms should be prioritised (i.e., ddPCR for rapid turnaround in routine monitoring, NGS in early-stage or exploratory settings where novel mutations and resistance mechanisms may be uncovered). Clear cut-offs validated in independent cohorts (stratified by geography and race) are critical for clinical adoption. While EVs show promise, isolation challenges currently limit their application; improved extraction technologies will be required before routine use.

Emerging biomarkers highlight the complexity of tumour immune interactions. Their diversity poses challenges in selecting the most clinically relevant markers, but also offers opportunities. With the advancement of deep learning methods, we can perform feature selection, aggregate high-dimensional data, and combine multiple biomarker layers into interpretable predictive multi-models.

Considering the financial budget allocated by governments to healthcare systems and the load of healthcare facilities, it is essential to evaluate factors such as turnaround time, test costs, false-positive rates, and the confidence each test provides in guiding clinical decisions. Additional considerations include the need for trained personnel to perform and interpret assays, and the potential to detect other diseases as the scope of testing expands. All these factors contribute to the failure of translating established biomarkers into routine clinical use for precision oncology.

The use of biomarker-guided adaptive treatment strategies is a growing trend in melanoma management [72,141,142,143]. This approach involves the employment of certain biomarkers for monitoring the treatment response and adjusting therapeutic plans from time to time. Beyond tailoring therapy during active treatment, such as switching drugs, adjusting dosage, or termination, this approach is also applicable to post-surgery scenarios. One example is the UK DETECTION trial, which aims to prevent overtreatment by initiating adjuvant therapy only after the detection of ctDNA relapse [73].

**Figure 2:**
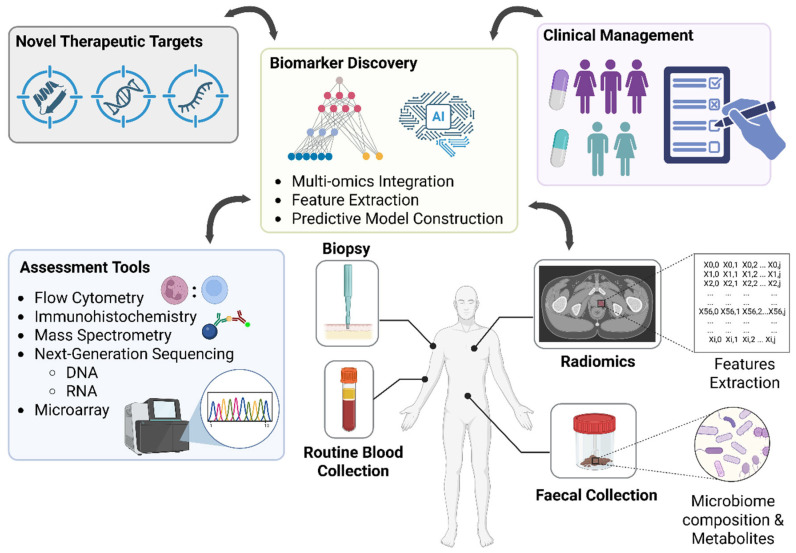
Practical framework illustrating the workflow from biomarker extraction through clinical implementation. In the exploratory stage, pilot studies employ a broad spectrum of assessment tools to capture molecular, clinical, and histopathological information. These multi-dimensional data are then integrated into deep-learning or machine-learning pipelines, where features are weighted not only by biological relevance but also by factors including cost, turnover time, robustness, and specificity. Filtered key features are subsequently used to construct AI-based predictive models, which are trained on existing datasets and ultimately implemented in routine clinical practice. Clinical outcomes can then feed back into the system to refine and boost model performance, while potentially uncovering hidden therapeutic targets for further investigation.

A practical framework for real-world implementation of biomarkers in melanoma should begin with assay standardisation across tissue, blood, and imaging platforms, supported by harmonised protocols and checklists. In terms of biomarker standardisation, the design of user-friendly commercial kits with a unified protocol, reagents, and scoring systems could help eliminate inter-laboratory variability. Clear guidelines for biomarker validation and benefit assessment, together with an organised online platform to help track tested biomarkers and their clinical contexts, would allow the gradual development of a comprehensive and consistent database. Standardisation of cut-offs and analytical methods can then be evaluated after the accumulation of validation trials under different independent cohorts. Automation can further strengthen this framework. Increasing the use of ML to handle repetitive processes would reduce human error and bias but enhance reproducibility. Composite risk models integrating clinical, histopathological, and molecular features, guided by AI-driven feature selection (Figure 2). Cost-conscious prioritisation, focusing on assays with rapid turnaround and scalability, recognising limited government healthcare budgets. Feedback loops in AI-driven predictive models, embedding multi-modal biomarkers into training pipelines, validating in independent cohorts, and iteratively refining performance based on real-world clinical outcomes.

Recent studies illustrate this potential: multi-omics AI models outperform TMB alone in predicting ICI response [144]; multimodal frameworks such as MelanoMAP integrate tumour microenvironment digital biomarkers with clinicopathological features to improve prognostication [145]; and AI-quantified TILs provide independent predictors of survival in ICI-treated patients [146].

Ultimately, the aim is to embed biomarker-guided decision-making into routine oncology practice, with dynamic, AI-assisted, and multi-layered strategies that capture tumour heterogeneity, guide adaptive therapy, and refine risk stratification. This will enable melanoma management to evolve from static guidelines towards truly personalised precision oncology, improving patient survival while reducing unnecessary toxicity and ordering effect. The MANIFEST platform (URL: https://www.manifest-io.org.uk) in the UK acts as one pilot initiative, providing a longitudinal sampling of multiple biospecimen types for detailed cellular and molecular profiling. The ultimate goal is to establish a quantitative, stratified tumour profile that can predict patient response to immunotherapy. Importantly, the platform also serves as a collaborative hub, bridging clinicians, researchers and industry partners to accelerate translation.

Another important note for improving the power of biomarkers in the future is to standardise the detection methods/platforms for biomarkers, which could facilitate eliminating the generation of inter-laboratory variations. This is crucial for interpreting, comparing, and setting the standard cut-offs for different biomarkers. Solutions to this note could include unifying protocols and reagents for assays, encouraging the employment of commercial kits (e.g., PD-L1 IHC 28-8 pharmDx assay as mentioned above), requiring certified detection equipment, and standard training for relevant personnel.

## 6. Conclusions

Predictive biomarkers of systemic therapy response in melanoma remain under active investigation, reflecting the complexity of tumour biology and immune interactions. While established markers such as *BRAF*, *NRAS*, and PD-L1 provide valuable insights, their predictive accuracy is limited (Table 1). Blood-based biomarkers, including LDH, S100B, and ctDNA, offer minimally invasive monitoring, with ctDNA particularly promising for tracking clonal evolution and distinguishing true progression from pseudoprogression. Emerging candidates, such as gene expression profiles, DNA methylation, miRNAs, mitochondrial genetics, IFN-γ signatures, and the gut microbiome, highlight the need for integrated, multi-parametric approaches.

Persistent challenges include assay variability, lack of standardisation, limited reproducibility, and translational barriers such as cost, turnaround time, and inconsistent cut-offs. Future progress will depend on automation, digitisation, and harmonisation of assays, alongside the adoption of multiplex and spatial technologies. Ultimately, dynamic AI-assisted composite frameworks that integrate tissue, blood, and imaging biomarkers will be essential to capture tumour heterogeneity, guide adaptive therapy, and refine risk stratification, advancing melanoma care toward truly personalised precision oncology.

## Figures and Tables

**Table 1:** Biomarkers in melanoma are stratified into tissue-based, blood-based, and emerging categories, with assessment tools, mechanisms, and predictive applications.

Type	Biomarkers	Assessment Tools	Mechanisms	Applications
Tissue-based	Gene alterations (*NRAS*, *EGFR*, *NF1*, *BRAF*, *PTEN*, *CCND1*, *CCKN2A*, *CDK4*, *TERT*)	NGS; qPCR or digital PCR; Sanger sequencing; FISH	MAPK pathway; PI3K/AKT pathway; Cell cycle control pathway; Telomere maintenance	Targeted therapy selection; ICI resistance prediction Prognostic stratification; Molecular subtyping
	TME (TILs, PD-L1 expression, TLS)	IHC; Flow Cytometry; RNA-sequencing; Spatial Transcriptomics	Immune evasion; Inflammation	Predict immunotherapy response
	TMB (High vs. Low)	WES or WGS; Large Targeted Panel	Neoantigen production; T-cell recognition	Predict immunotherapy response
Blood-based	High LDH	Colorimetric assays; Fluorometric assays; Bioluminescent assays	Warburg effect; Immunosuppressive environment	Assess immune cell-induced cytotoxicity; Reflect TMB and tumour aggressiveness
	S100B	ELISA	RSK/ERK pathway; p53 pathway; IL6/STAT3 signalling	Melanoma specificity; Disease progression
	ctDNA	ddPC; NGS	Tumour shedding	Real-time monitor; Tumour heterogeneity; MRD detection
	EVs	Ultracentrifugation; Precipitation kits; Size exclusion chromatography	tumour immune cell bridging; Metastasis promotion; Immune modulation; Resistance-associated molecules	Predict anti-PD-1 therapy response; Real-time monitoring
	Elevated osteopontin levels	ELISA	Angiogensis; Lymphangiogenesis; Immune evasion	Serial monitoring; Prognostic prediction
Emerging	Immune transcriptome (Cytokines, INF alpha)	RNA-sequencing; qRT-PCR; Multiplex immunoassays; Flow cytometry; IHC	JAK-STAT pathway; Angiogenesis; Immune modulation; tumour immune interactions	Serial monitoring; Prognostic prediction
	DNA methylation	Microarray; Bisulfite sequencing; Pyrosequencing	Tumour suppressor silencing; Immune modulation; Therapy resistance	ctDNA methylation monitoring; Therapy resistance prediction
	miRNA Non-coding RNA	Microarray; RNA-sequencing; qPT-PCR	Drive drug resistance; Gene regulation	Prognostic prediction; Serial monitoring
	Mitochondrial DNA	NGS; qPCR or digital PCR; ddPCR	Haplogroups; Copy number alternations; Metabolic rewiring	Mechanistic investigation
	Gut Microbiome	16s rRNA sequencing; Shotgun metagenomic sequencing; Metabolomics; Metatranscriptomics	Immune modulation; Metabolite signalling; Host–tumour interactions	Predict immunotherapy response
	Radiomics	CT; MRI; PET		Non-invasive monitoring; Prognostic prediction; Organ-specific resistance; TME reflection

TIL, tumour-infiltrating lymphocytes; TLS, tertiary lymphoid structures; TMB, tumour mutational burden; ctDNA, circulating tumour DNA; EV, extracellular vesicles; miRNA, microRNA; NGS, next-generation sequencing; qPCR, quantitative polymerase chain reaction; FISH, fluorescence in situ hybridisation; IHC, immunohistochemistry; WES, whole-exome sequencing; WGS, whole-genome sequencing; ELISA, enzyme-linked immunosorbent assay; ddPCR, droplet digital PCR; qRT-PCR, quantitative reverse transcription PCR; CT, computed tomography; MRI, magnetic resonance imaging; PET, positron emission tomography.

## Data Availability

No new data were created or analysed in this study. Data sharing is not applicable.
